# Supplementary material for: Translational reprogramming as a driver of antimony-drug resistance in Leishmania
Source: Nat Commun. 2023 May 5;14:2605. doi: 10.1038/s41467-023-38221-1 (PMC10163012; doi:10.1038/s41467-023-38221-1)
Supplement: Supplementary file 1 — Supplementary Information file [file 41467_2023_38221_MOESM1_ESM.pdf]

Supplementary Information files for:

Translational Reprogramming as a Driver of Antimony-drug Resistance in *Leishmania*

Sneider Alexander Gutierrez Guarnizo [1,2], Elena B. Tikhonova [1], Andrey L. Karamyshev [1,4], Carlos E. Muskus [2,4], and Zemfira N. Karamysheva[3,4]

[1] Department of Cell Biology and Biochemistry, Texas Tech University Health Sciences Center, Lubbock, TX, 79430, USA.

[2] Programa de Estudio y Control de Enfermedades Tropicales. Universidad de Antioquia. Medellín 050010, Colombia

[3] Department of Biological Sciences, Texas Tech University, Lubbock, TX, 79409, USA.

[4] These authors contributed equally to this work.

Corresponding authors

Zemfira N. Karamysheva

zemfira.karamysheva@ttu.edu (Z.N.K.), Tel.: +1-806-834-5075.

Carlos E. Muskus

carlos.muskus@udea.edu.co (C.M.), Tel.: +57-300-618-4852.

Andrey L. Karamyshev

andrey.karamyshev@ttuhsc.edu (A.L.K.), Tel.: +1-806-743-4102.

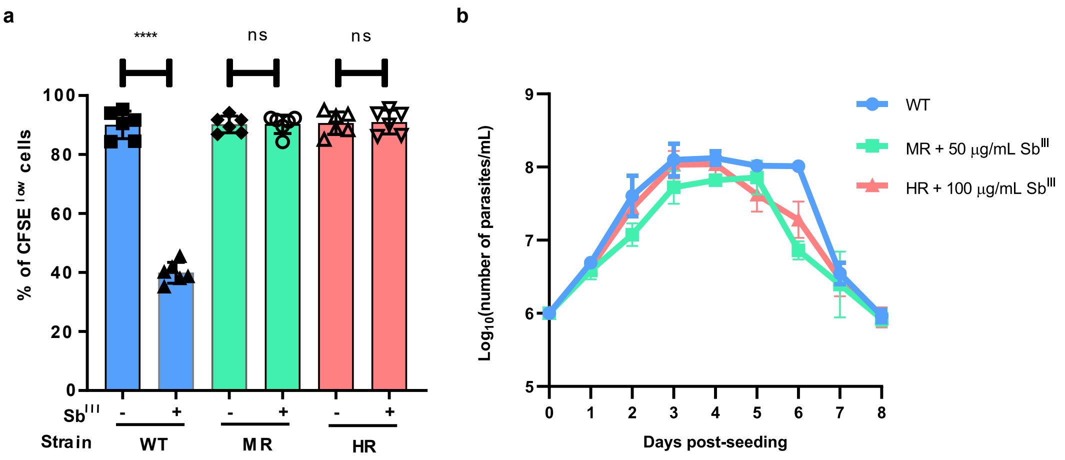

Supplementary Figure 1. Sb^{III}-resistant parasites do not show significant changes in proliferation when compared to the parental-sensitive strain. (a) Parasites stained with 1 µM CellTrace™ CFSE for cell proliferation assay were seeded at 1X10^{6} parasites/mL. After 3 days of growth, the percentage of parasites with CFSE signal decreased (CFSE^{low}) was estimated as a measure of active proliferation. Parasites were treated (+) or not (-) with 20 µg/mL (2X the EC50 for WT strain). The sensitive (WT) but not the resistant strains (MR and HR) showed reduced proliferative capability in presence of the drug. One-way ANOVA test. F score: 185.7. Degree of freedom numerator: 5. Degree of freedom denominator: 30. Multiple comparisons analysis by Tukey test. P-value: 4.6X10^{-14} (****). P-value: 0.99 (ns). n: 6 biologically independent replicates. Data are presented as mean values +/- SD. **(b)** Parasites seeded at 1X10^{6} parasites/mL were counted daily by the Neubauer chamber. The X-axis represents the days post-seeding. Y-axis summarizes the log-transformed number of parasites per milliliter (mL). Resistant parasites cultured under drug pressure show a similar growth profile when compared to the untreated WT strain (control). n: 3 biologically independent replicates. Data are presented as mean values +/- error with 95% CI.

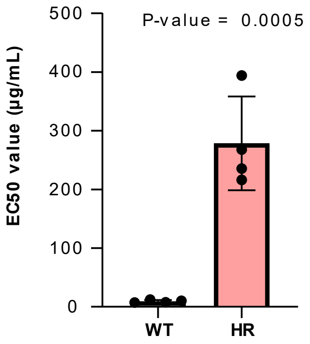

Supplementary Figure 2. The HR strain displays a stable resistant phenotype after multiple passages without drug challenge. *Leishmania tropica* highly resistant strain (HR) was examined for its ability to maintain antimony resistance in the absence of the drug. Wild-type (WT) and HR strains were re-seeded 25 times in drug-free conditions. The EC50 value was re-measured again by MTT assay using four biologically independent replicates with three technical repeats for each strain. Statistical analysis was performed using a two-tailed unpaired t test. t distribution value: 6.728, degrees of freedom: 6. Data are presented as mean ± SD.

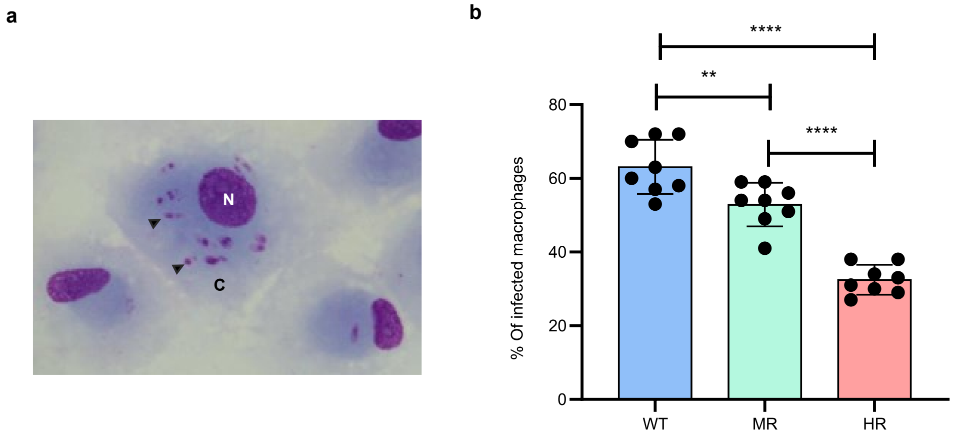

Supplementary Figure 3. The stepwise selection for Sb^{III}-resistance is associated with a progressive decrease in the infectivity of parasites. (a) Representative picture of phagocytic cell derived of U-937 monocytes infected by *L. tropica* (HR strain). Cytosol (C). Nucleous (N). Intracellular amastigotes are highlighted by arrows. **(b)** Percentage of phagocytic cells infected by WT, MR, and HR strains. Ordinary one-way ANOVA. F distribution value: 55.04. Degrees of freedom numerator: 2. Degrees of freedom denominator: 3. Multiple comparisons performed by Tukey test. P-value (WT vs. MR): 6.51×10^{-03}. P-value (WT vs. HR): 3.35×10^{-09}, P-value (MR vs. HR): 2.58×10^{-06}. P-value 0.001 to 0.01 (**). P-value < 0.0001 (****). n: 8 biologically independent replicates. Data are presented as mean values +/- SD.

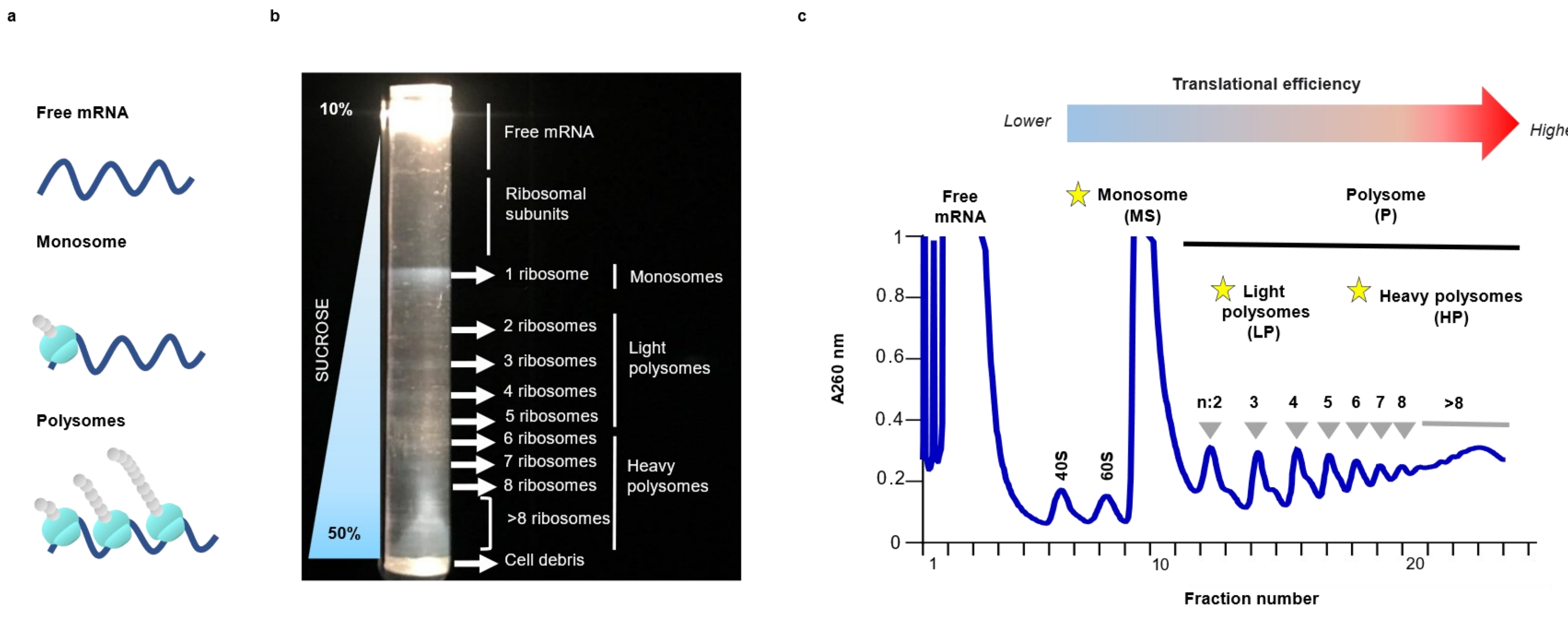

Supplementary Figure 4. Polysome profiling for translatome analysis. The number of ribosomes interacting with an mRNA is an indicator of its translational efficiency. **(a)** Graphical representation of different types of mRNA in terms of their translational status. Free mRNA is an mRNA that is not engaged with ribosomes and consequently, it is not being translated. A monosome is a single mRNA associated with one ribosome, so it is assumed to be under translation. A polysome corresponds to a single mRNA engaged with two or more ribosomes. As more ribosomes are coupled to a single transcript, the translation is more efficient, and the complex is heavier. **(b)** 10-50% sucrose gradient of *L. tropica* promastigotes lysate after ultra-centrifugation. Monosomes (mRNA interacting with a single ribosome), light polysomes (mRNA interacting with 2-5 ribosomes), and heavy polysomes (mRNA interacting with 6 or more ribosomes) are observed from the top to the bottom of the tube. **(c)** Polysome profiling plot built after automatic sucrose gradient polysome fractionation showing the RNA distribution as the absorbance at 260 nm (Y-axis) versus the polysome fraction (X-axis).

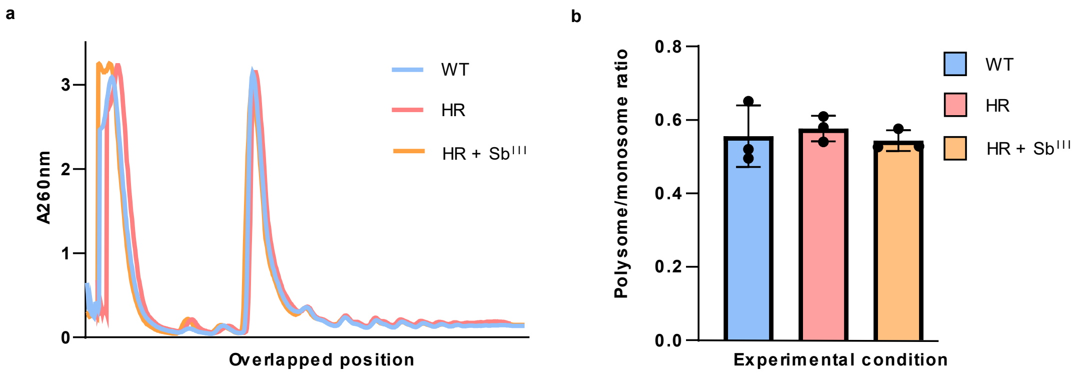

Supplementary Figure 5. Polysome profiles of antimony-resistant and sensitive parasites show similar patterns. To estimate changes in translational capacity, the polysome profiles were compared among all experimental conditions. **(a)** overlapping of polysome profiles from sensitive strain (WT), resistant strain (HR), and resistant strain growing under drug challenge (HR+Sb^{III}). **(b)** Comparison of polysome profiles based on polysome-to-monosome (P/M) ratio. The areas under the curve for polysomes and monosomes were estimated using the ImageJ 1.8 software. Statistical analysis was performed by a one-way ANOVA analysis. F distribution value: 0.2783. Degrees of freedom for the numerator: 2. Degrees of freedom for the denominator: 6. P-value: 0.7663. n:3 biologically independent replicates. Data are presented as mean values +/- SD.

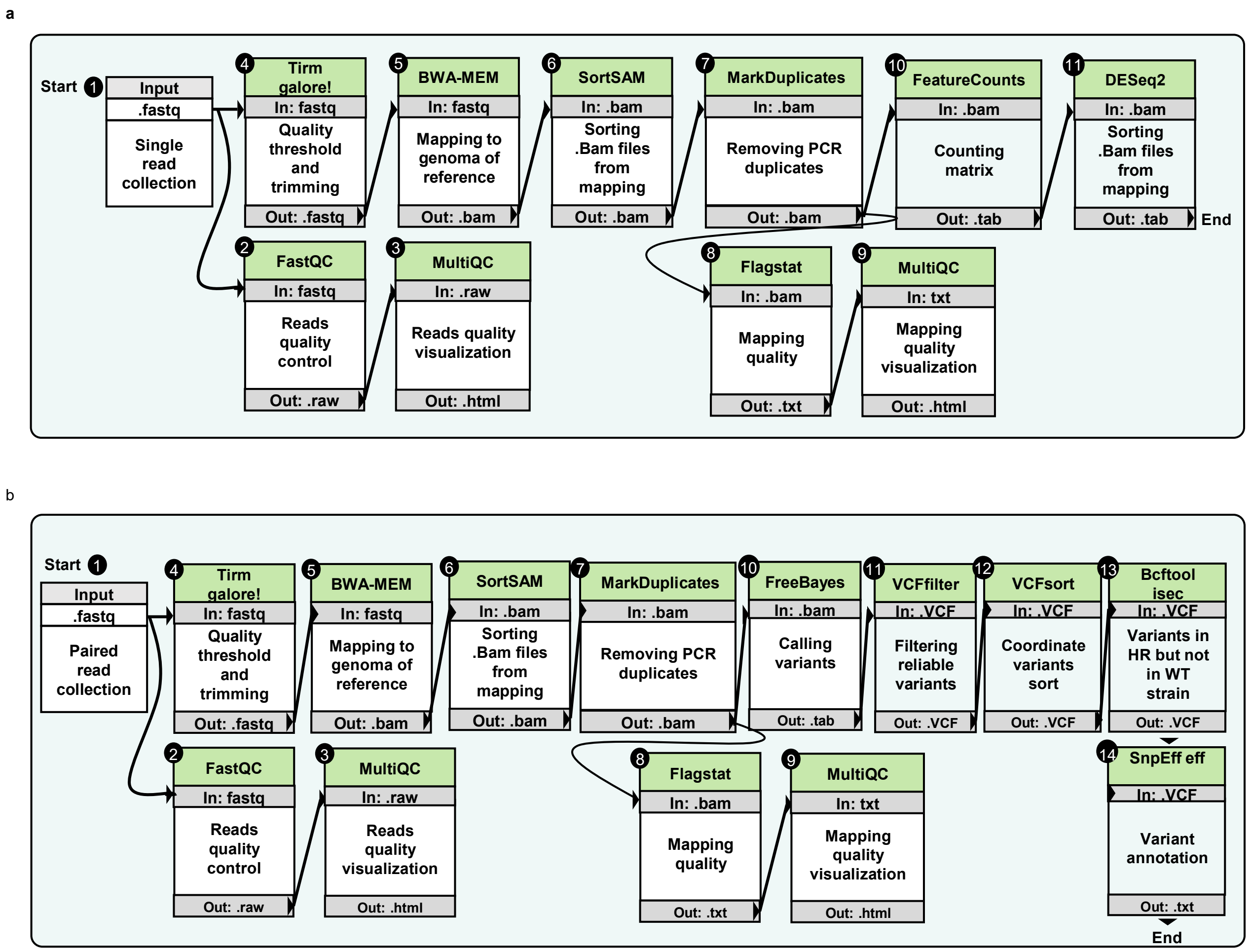

Supplementary Figure 6. Strategy for bioinformatic analysis. Bioinformatic analysis was performed using Galaxy server as independent reproducible workflows. Each individual box represents a different step in the workflow. The number on the left top of each box represents the

logical order. The algorithm used per step is highlighted in green color. The purpose of each step is described in the center of the boxes. The corresponding format of the input (In) and output files (Out) are annotated in grey boxes. The arrows connect each output with the next step. **(a)** Bioinformatic workflow for DTA analysis. The samples per experimental condition were run as independent data collection until creating the respective counting matrix (step 10). Then, the DTA analysis was based on the DESeq2 algorithm by dual comparisons of the respective counting matrices (step 11). **(b)** Bioinformatic workflow for variant calling analysis. The samples per experimental condition (WT and HR) were run as independent data collection until the coordinated sort of the.VCF files (step 12). Then, the lists of variants were compared to identify the variants exclusively present in HR strain (step 13), followed by variant annotation (step 14).

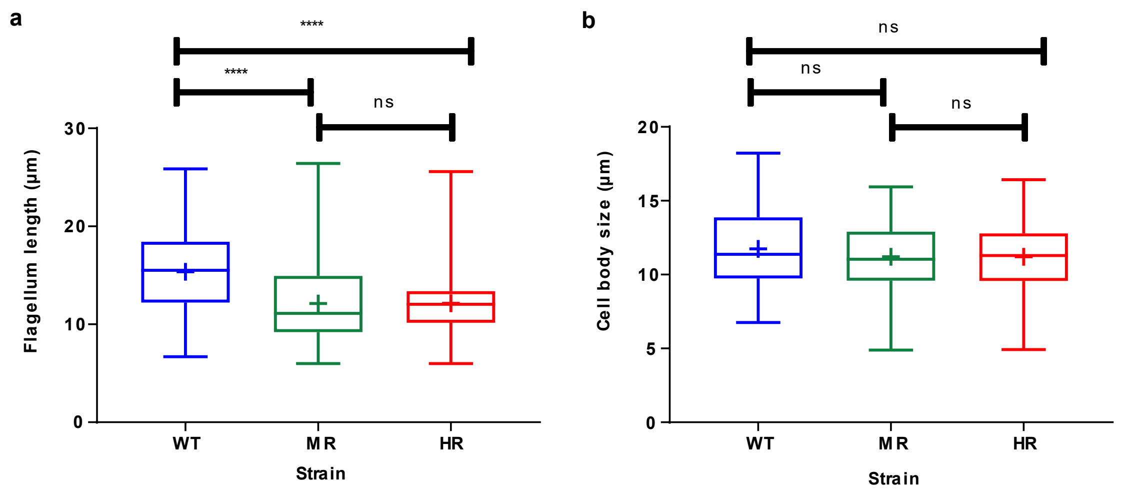

Supplementary Figure 7. Sb^{III}-resistant parasites showed smaller flagellum but no differences in cell body length. **(a)** comparison of *L. tropica's* flagellum size among Sb^{III}-sensitive (WT), and Sb^{III}-resistant parasites (MR and HR). Number of groups: 3. Right tailed Kruskal-wallis statistic: 43.63. Multiple comparison corrected by Dunn's test. P-value < 0.0001 (***). P-value > 0.9999 (no significant, ns). P-value (WT vs. MR): 1.3X10^{-8}. P-value (WT vs. HR): 8.2X10^{-8}, P-value (MR vs. HR): 0.99. n: = 100 parasites examined over 3 independent experiments. **(b)** comparison of *L. tropica's* cell body among Sb^{III}-sensitive (WT), and Sb^{III}-resistant parasites (MR, HR). One way ANOVA test. F distribution value: 1.929. P-value: 0.1470. Numerator degrees of freedom: 2. Denominator degrees of freedom: 326. n: = 100 parasites examined over 3 independent experiments. **(a,b)** The box bounds the IQR divided by the median, and Tukey-style whiskers extend to a maximum of 1.5 × IQR beyond the box. WT, MR, and HR strains were treated with 0, 50 and 100 µg/mL for 48 hours.

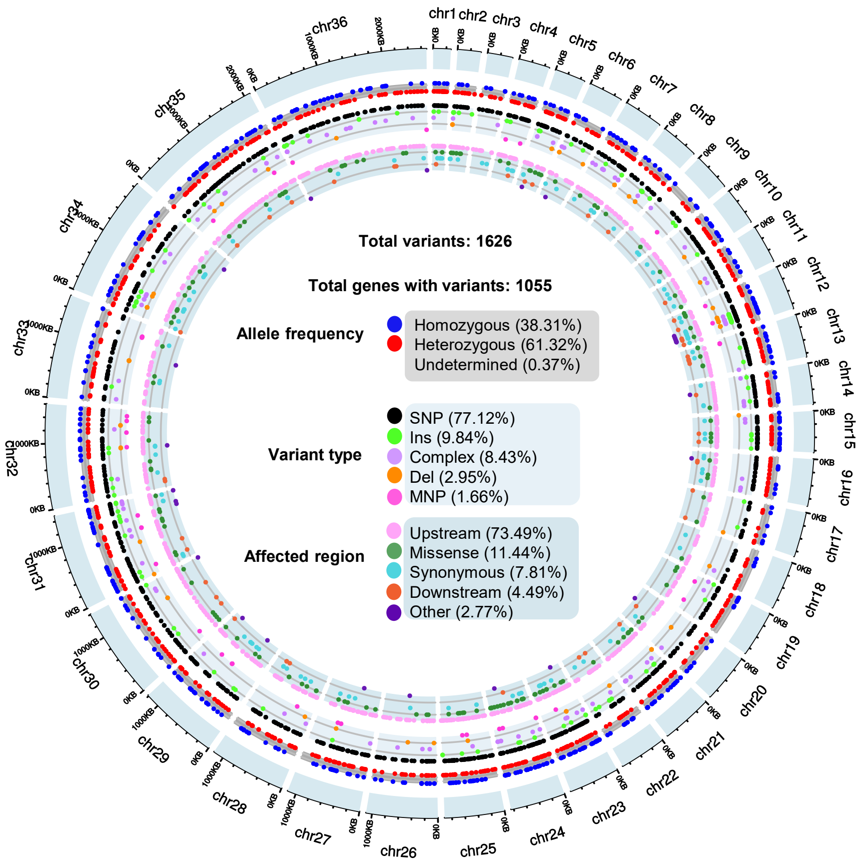

Supplementary Figure 8. Most of the variants detected in Sb^{III}-resistant strain are heterozygous SNPs located upstream of genes. Circus plot showing 4 different tracks. From the outer to the inner circle: distribution of chromosome (chromosomes 1-36), allele frequency (homozygous or heterozygous), variant type (single nucleotide polymorphism or SNP, insertion, or Ins, complex, deletion or Del, multiple nucleotide polymorphism or MNP), and variant region (upstream, missense, synonymous or downstream). The dots represent each individual variant mapped to the genome reference. The color described in the center of the plot indicates the variant classification.
