# Supplementary material for: Translational reprogramming as a driver of antimony-drug resistance in Leishmania
Source: Nat Commun. 2023 May 5;14:2605. doi: 10.1038/s41467-023-38221-1 (PMC10163012; doi:10.1038/s41467-023-38221-1)
Supplement: Supplementary file 3 — Description of additional supplementary files [file 41467_2023_38221_MOESM3_ESM.pdf]

Translational Reprogramming as a Driver of Antimony-drug Resistance in *Leishmania*

Supplementary files description:

File name: *Supplementary Data 1*

Description: Raw data of automatic polysome fractionation per experimental condition.

File name: *Supplementary Data 2:*

Description: Differentially expressed genes detected in antimony-resistant parasites at the basal level (no drug challenge).

File name: *Supplementary Data 3:*

Description: Raw data from Venn diagram summarizing the differentially expressed genes detected at the basal level (no drug challenge) per polysome fraction.

File name: *Supplementary Data 4:*

Description: Comparison of the translatome Vs. transcriptome (basal level).

File name: *Supplementary Data 5:*

Description: Dataset of differentially expressed genes detected in antimony resistant parasites growing under drug challenge.

File name: *Supplementary Data 6:*

Description: Raw data from Venn diagram summarizing the differentially expressed genes detected in antimony resistant parasites growing under drug challenge per polysome fraction.

File name: *Supplementary Data 7:*

Description: Comparison of the translatome Vs. transcriptome (drug challenge).

File name: *Supplementary Data 8:*

Description: Dataset summarizing the normalized read counts by DESeq2's median of ratios.

File name: *Supplementary Data 9:*

Description: List of primers used for the validation of translatome analysis by RT-qPCR.

File name: *Supplementary Data 10:*

Description: Comparison between translatomic and proteomic analyses for resistant parasites growing under drug challenge.

File name: *Supplementary Data 11:*

Description: Dataset summarizing known and potentially new antimony modulators

File name: *SupplementaryData 12:*

Description: Raw data of gene ontology enrichment analysis.

File name: *Supplementary Data 13:*

Description: Clustering analysis based on protein homology summarizing changes at the basal level (no drug challenge).

File name: *Supplementary Data 14:*

Description: Clustering analysis based on protein co-expression summarizing changes at the basal level (no drug challenge).

File name: *Supplementary Data 15:*

Description: Raw data for the glutathione metabolism STRING network dataset summarizing changes under drug challenge.

File name: *Supplementary Data 16:*

Description: Manually grouped differentially translated transcripts detected in antimony resistant parasites growing under drug challenge.

Supplementary Data 17:

Description: Gene variants detected in antimony resistant parasites by using the FreeBayes algorithm.

Supplementary Data 18:

Description: Comparison between translatomic and variant calling analyses.
